# Variation in transcriptional regulation of cyclin dependent kinase inhibitor p21^waf1/cip1 ^among human bronchogenic carcinomas

**DOI:** 10.1186/1476-4598-4-23

**Published:** 2005-07-13

**Authors:** Michael W Harr, Timothy G Graves, Erin L Crawford, Kristy A Warner, Cheryl AM Reed, James C Willey

**Affiliations:** 1 Department of Medicine, Medical University of Ohio, 219 Health Education Building, 3055 Arlington Avenue, Toledo, OH, 43614-5806, USA; 2 Department of Cariology, Restorative Sciences and Endodontics, University of Michigan, 2310A Dental Research Building, 1011 North University Avenue, Ann Arbor, MI, 48109-1078, USA

## Abstract

**Background:**

Cell proliferation control depends in part on the carefully ordered regulation of transcription factors. The p53 homolog p73, contributes to this control by directly upregulating the cyclin dependent kinase inhibitor, p21^waf1/cip1^. E2F1, an inducer of cell proliferation, directly upregulates p73 and in some systems upregulates p21 directly. Because of its central role in controlling cell proliferation, upregulation of p21 has been explored as a modality for treating bronchogenic carcinoma (BC). Improved understanding of p21 transcriptional regulation will facilitate identification of BC tissues that are responsive to p21-directed therapies. Toward this goal, we investigated the role that E2F1 and p73 each play in the transcriptional regulation of p21.

**Results:**

Among BC samples (N = 21) p21 transcript abundance (TA) levels varied over two orders of magnitude with values ranging from 400 to 120,000 (in units of molecules/10^6 ^molecules β-actin). The p21 values in many BC were high compared to those observed in normal bronchial epithelial cells (BEC) (N = 18). Among all BC samples, there was no correlation between E2F1 and p21 TA but there was positive correlation between E2F1 and p73α (p < 0.001) TA. Among BC cell lines with inactivated p53 and wild type p73 (N = 7) there was positive correlation between p73α and p21 TA (p < 0.05). Additionally, in a BC cell line in which both p53 and p73 were inactivated (H1155), E2F1 TA level was high (50,000), but p21 TA level was low (470). Transiently expressed exogenous p73α in the BC cell line Calu-1, was associated with a significant (p < 0.05) 90% increase in p21 TA and a 20% reduction in E2F1 TA. siRNA mediated reduction of p73 TA in the N417 BC cell line was associated with a significant reduction in p21 TA level (p < 0.01).

**Conclusion:**

p21 TA levels vary considerably among BC patients which may be attributable to 1) genetic alterations in *Rb *and p53 and 2) variation in TA levels of upstream transcription factors E2F1 and p73. Here we provide evidence that p73 upregulates p21 TA in BC tissues and upregulated p21 TA may result from E2F1 upregulation of p73 but not from E2F1 directly.

## Background

Cell cycle homeostasis in normal human bronchial epithelial cells (BEC) is highly regulated at the G1/S transition. In G1 phase of the cell cycle, formation of a heterodimeric complex between cyclin D and cyclin dependent kinases 4 or 6 (cdk 4,6) leads to the phosphorylation of the tumor suppressor retinoblastoma gene product (pRb) [1-3]. Phosphorylation causes conformational change of the pRb/E2F complex, followed by release, and activation of the E2F1, 2, and 3 transcription factors [4-6]. Free E2F proteins bind strongly to DNA and were first identified by their ability to transactivate the adenoviral E2 promoter [7]. E2F1 functions to upregulate transcription of genes required for entry into S phase, including cyclin E, *c-myc *and itself [8-10]. In turn, c-*myc *directly upregulates transcription of cyclin E and cdk4 [11,12]. Thus, phosphorylation of pRb by one or more cyclin/cdk complexes causes activation and upregulation of E2F1, upregulation of c-*myc *transcription by E2F1, and upregulation of cdk4 transcription by c-*myc*. These interactions are initiated at the restriction point of G1/S, which is associated with independence of the cell from extracellular growth factors [4,13]. The events described above contribute to a cell proliferation signal amplification cycle that would be uncontrolled in the absence of compensatory negative feedback.

Compensatory feedback signals, including the activation of p53 and transcriptional upregulation of p73 and p21^waf1/cip1 ^(p21 hereafter) act to slow cell proliferation [14-16]. Unlike p53, p73 is not frequently mutated in human cancers [17], and thus it is not considered a classical tumor suppressor gene, as defined by Knudson's two hit hypothesis [18,19]. However, it functions to promote cell cycle arrest, DNA repair, and apoptosis much like p53 [18,20]. E2F1 (and c-*myc*) transactivation of p14^ARF ^leads to stabilization of p53 [21,22] which slows cell cycling through the upregulation of p21 [23,24], and also induces apoptosis [25]. E2F1 also upregulates p73 [15,26,27] and p73 upregulates p21 [15], which in turn acts to inhibit the release of E2F1 from pRb, resulting in compensatory feedback for the loss of cell proliferation control. In some systems, E2F1 also upregulates p21 directly [28].

In previous studies, we determined that p21 transcript abundance (TA) levels vary considerably among bronchogenic carcinoma (BC) primary tissues and cell lines, and in some of these samples the p21 TA level is higher than the level observed in normal BEC [29,30]. Because p21 normally slows cell proliferation, this observation was unexpected, and counter-intuitive. If validated, these preliminary findings would have important implications for the many efforts presently underway to design gene specific cancer treatments that function to achieve cell proliferation control [31].

The purpose of this study was to better understand inter-tumor variation in the mechanisms responsible for loss of proliferation control and to better define the role of E2F1, p73 and p21 regulatory pathways as they relate to cell proliferation control in human BC. Our approach included additional descriptive studies in BC cell lines, as well as primary BC tissues and normal BEC to better quantify inter-tumor variation of p21 TA levels. With respect to the fourteen BC cell lines used in this study, because each has been extensively characterized at the genetic level [17,25,32-35], we were better able to define the regulatory relationship between E2F1, p73, and p21 after considering the known alterations in each individual cell line. Our descriptive approach was followed by experimental testing of the hypothesis posed to explain these observations.

## Results

Transcript abundance (TA) levels of E2F1, p73α, and p21 in normal bronchial epithelial cells (BEC) (N = 18) and bronchogenic carcinoma (BC) tissues (N = 21) were measured by StaRT-PCR. Cultured cells and primary tissues are shown in Tables 1 and 2, respectively. For the BC cell lines, known alterations in p53, p16, p14^ARF^, *Rb*, p73, and *c-myc *also are presented, along with the TA values. Bivariate analysis of the TA data from these cultured human BEC samples provided data consistent with observations in other tissues that E2F1 regulates p73 transcription [15,26,27] and that p73 regulates p21 transcription [15]. However, in contrast to other cell types, they suggested that E2F1 does not directly regulate p21 transcription. To further investigate the significance of this finding, seven primary BC tissues (Table 2) were also assessed.

**Table 1:** Transcript abundance measurements for cultured normal BEC and BC cell lines

**Sample**	**Cell Type**	**Medium**	**Genetic Alterations**						**Transcript Abundance**		
			**p53**	**p16**	**p14^ARF^**	***Rb***	**p73**	***c-myc***	**E2F1**	**p73α**	**p21**

17378B	N	B							1,500	1	21,000
6F0333B	N	B							4,700	1	76,000
6F0395B	N	B							5,100	1	120,000
SW900	Sq	R		I					7,300	100	12,000
Calu-1	Sq	R	I	I					8,500	28	2,000
H460	LC	R		I					9,000	14	18,000
H82	SC	R	I		I	I		A	15,000	17	400
A549	A	R		I	I				16,000	1	6,200
A2126	A	R	I						17,000	30	6,100
N417	SC	R	I			I		A	19,000	5,200	34,000
H322	BA	R	I	I					20,000	190	31,000
H446	SC	R				I		A	67,000	710	120,000
H1155*	LC	R	I			I	I		50,000	250	470
H520	Sq	R	I	I					56,000	120	2,300
H146	SC	R				I			59,000	700	19,000
H661	LC	R	I	I					76,000	210	58,000
A427**	A	R		I					180,000	220	100,000

N, normal; Sq, squamous carcinoma; LC, large cell carcinoma; SC, small cell carcinoma; A, adenocarcinoma; BA, bronchoalveolar carcinoma; B, BEGM medium; R, RPMI with 10% FBS; I, inactivated; A, amplification. Non-detectable TA were entered as 1 to allow plotting on a logarithmic scale. Cell lines are grouped by increasing E2F1 TA. All values are in units of molecules/10^6 ^molecules β-actin and represent the mean from three independent measurements. *H1155 has a missense mutation in the transactivation domain of p73. **A427 has a deletion in the p73 coding sequence but the protein product still retains its transactivational function [35].

**Table 2:** Transcript abundance measurements for primary normal BEC and BC tissues

**Sample ID**	**Diagnosis**	**Cell Type**	**E2F1**	**p73α**	**p21**
63	NC	N	270	850	14,000
282	NC	N	540	640	10,000
64	NC	N	2,000	400	10,000
285	NC	N	2,500	870	3,100
257	NC	N	270	5,200	12,000
156	NC	N	750	1,000	25,000
194	NC	N	290	3,800	3,500
150	NC	N	160	3,700	11,000
136	NC	N	700	31	9,100
261	NC	N	250	3,100	30,000
191	C	N	150	900	13,000
158	C	N	190	2,200	11,000
146	C	N	230	2,600	9,100
34	C	N	570	4,400	12,000
212	C	N	600	5,300	35,000
274	C	Sq	730	1	7,000
279	C	A	1,200	1	4,000
190	C	BA	6,600	3	27,000
102	C	A	12,000	21	21,000
165	C	SC	36,000	4,300	16,000
123	C	Sq	54,000	5,500*	90,000
277*	C	A	120,000	100	2,000

NC, non-cancer; C, cancer; N, normal; Sq, squamous carcinoma; A, adenocarcinoma; BA, bronchoalveolar carcinoma; SC, small cell carcinoma. Non-detectable TA were entered as 1 to allow plotting on a logarithmic scale. Normal BEC are grouped by diagnosis and cell type. BC tissues are grouped by increasing E2F1 TA. All values are in units of molecules/10^6 ^molecules β-actin and represent the mean from three independent measurements. *This value represents the mean from two measurements due to insufficient sample.

The mean, median, and quartile values for each gene in normal (N = 18) compared to malignant samples (N = 21) are shown in Table 3. E2F1 TA levels were over 30-fold higher in BC relative to normal BEC (p < 0.0001). Conversely, mean p73α TA was higher in normal BEC by over 2-fold, but this difference was not statistically significant (p = 0.07), although the median p73 TA value was higher in normal BEC by nearly 9-fold. With respect to p21 TA level, there was no statistical difference in the mean value between the two groups.

**Table 3:** Descriptive statistics for E2F1, p73α, and p21 transcript abundance measurements

**Normal BEC**				**Bronchogenic Carcinoma**			
	**E2F1**	**p73α**	**p21**		**E2F1**	**p73α**	**p21**

**Mean**	**1,200**	**1,900**	**24,000**	**Mean**	**40,000**	**840**	**25,000**
Min	150	1	3,100	Min	730	1	400
LQ	260	460	10,000	LQ	9,000	17	4,000
**Median**	**560**	**950**	**12,000**	**Median**	**19,000**	**100**	**12,000**
UQ	1,300	3,600	24,000	UQ	56,000	250	27,000
Max	5,100	5,300	120,000	Max	180,000	5,500	120,000

Min, minimum; LQ, lower quartile; UQ, upper quartile; max, maximum. Values were derived from statistical analysis of samples presented in Tables 1 and 2.

Median and quartile values were used to determine if TA levels were high or low for a given sample. For example, the median value for E2F1 in BC was 19,000. Therefore, a sample with an E2F1 TA level greater than 19,000 would be considered high and a TA level less than 19,000 would be considered low. These criteria were used to identify cell lines with low (Calu-1) or high (N417) p73α TA for use in the exogenous p73 and p73 siRNA transfection experiments.

### Bivariate analysis of E2F1 and p21

**Figure 1:**
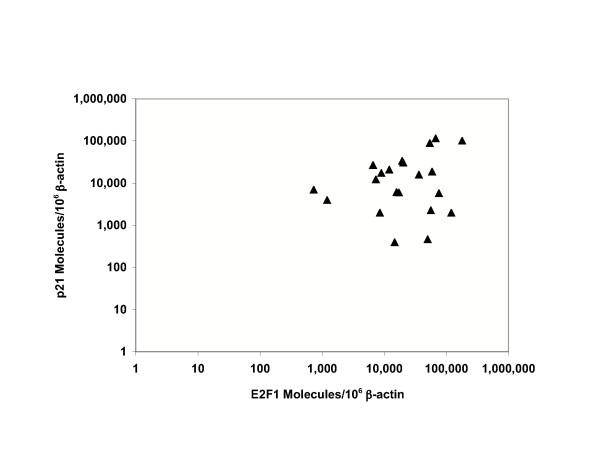
**Lack of correlation between E2F1 and p21 TA**. There was no correlation between E2F1 and p21 TA among BC cell lines (N = 14) and primary BC tissues (N = 7). Each point represents the mean value from triplicate measurements of E2F1 and p21 as shown in Tables 1 and 2 (except where indicated).

There was no significant correlation between E2F1 and p21 TA levels among BC cell lines and primary tissues (N = 21) (Figure 1).

### Bivariate analysis of E2F1 and p73α TA levels

**Figure 2:**
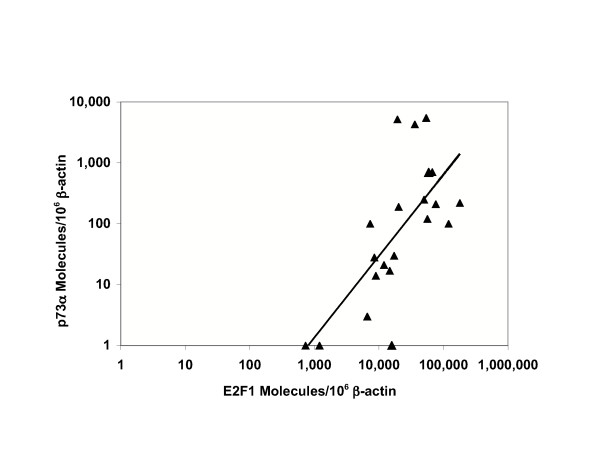
**Bivariate analysis of E2F1 and p73α TA**. E2F1 and p73α TA were positively correlated among BC cell lines (N = 14) and primary BC tissues (N = 7). Each point represents the mean value from triplicate measurements of E2F1 and p73α as shown in Tables 1 and 2 (except where indicated).

E2F1 and p73α TA values were positively correlated (p < 0.001) among BC cell lines and primary tissues (N = 21) (Figure 2).

### Bivariate analysis of p73α and p21 TA levels

**Figure 3:**
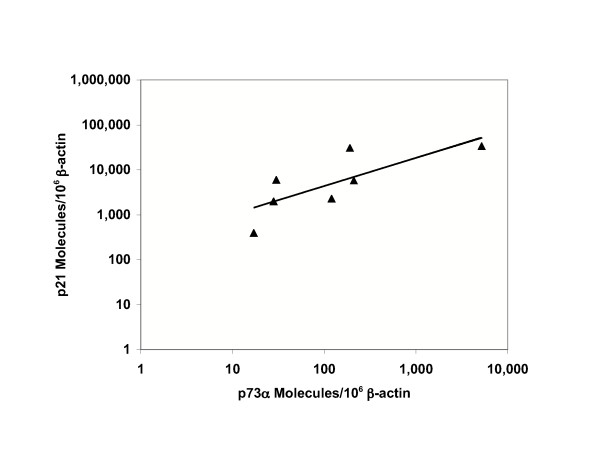
**Bivariate analysis of p73α and p21 TA**. p73α and p21 TA were positively correlated (p < 0.05) among BC cell lines (N = 7) where p53 was known to be inactivated by mutation or deletion and p73 was wild type. Each point represents the mean value from triplicate measurements of p73α and p21 as shown in Table 1.

Among BC cell lines in which p53 is known to be completely inactivated by mutation or deletion (N = 7), p73α and p21 were significantly correlated (p < 0.05), as shown in Figure 3. In contrast, there was a borderline but insignificant (p = 0.06) positive correlation between p73α and p21 TA among all BC cell lines and primary tissues (N = 21). This result may be explained by p53 regulation of p21 transcription as a confounding variable in those cell lines with wild type p53.

### E2F1 and p21 TA analysis in BC cell line with inactivated p53 and p73

In the H1155 cell line in which p53 and p73 are both inactivated, E2F1 TA level was high, yet p21 TA level was low (Table 1). Consistent with our observation that E2F1 and p73α are correlated in BC tissues (Figure 2), E2F1 and p73α TA levels were both high in H1155. However, the missense mutation in the DNA binding domain of p73 inhibits its transactivational function [35]. Thus, H1155 is a cell line with a naturally occurring p73 mutation that directly supports our hypothesis that p21 is not upregulated by E2F1 directly.

### Expression of exogenous p73α is associated with increased p21 and decreased E2F1 TA in Calu-1

**Figure 4:**
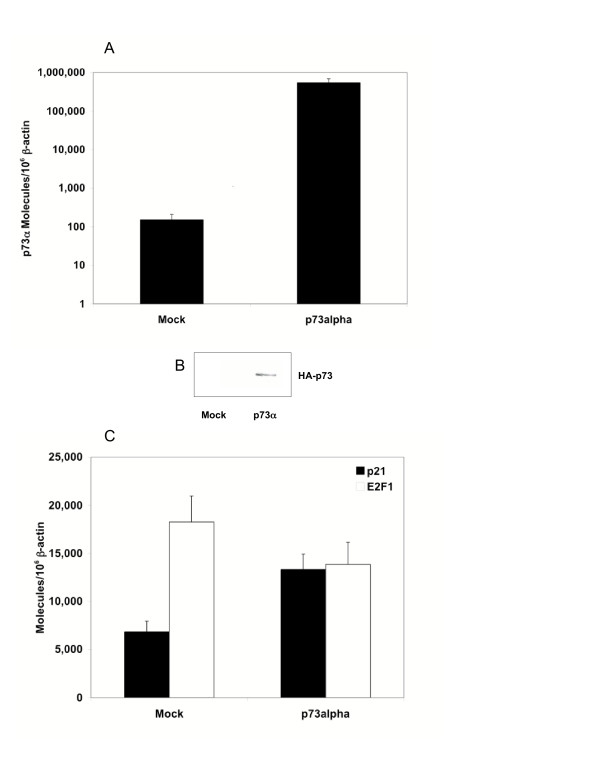
**Effect of p73α transient expression on p73α, p21, and E2F1 TA in Calu-1 cells**. **A) **p73α TA was induced by over 3 orders of magnitude relative to mock transfected cells. Calu-1 cells were transfected with 5 μg of GFP control plasmid or HA-p73α. **B) **Total HA-p73 protein was analyzed in mock or p73α transfected cells. 20 μg of lysate were blotted on a PDVF membrane and incubated with an anti-hemagglutinin primary antibody conjugated to HRP. **C) **p21 was upregulated by 90% and E2F1 was reduced by 20% in p73α transfected cells. Results represent the mean value from triplicate measurements from three independent experiments. Error bars represent the S.E.M. RNA was extracted 24 hours post-transfection and treated with DNaseI. RNA was PCR amplified to rule out the possibility of plasmid contamination. No PCR products were detected.

To test the hypothesis that p21 transcription is regulated by p73 and not by E2F1 directly, p73α was transiently expressed in the squamous carcinoma cell line Calu-1. This line expresses low levels of endogenous p73α TA (28), low levels of p21 TA (2,000), and low levels of E2F1 TA (8,500). p73α TA was induced over 1,000-fold relative to the mock, 24 hours post-transfection (Figure 4A). Exogenous p73 protein expression was confirmed by Western analysis using an anti-HA antibody specific for an amino terminal tag on p73 (Figure 4B). p21 and E2F1 TA levels were quantified 24 hours post-transfection. While p21 TA was upregulated 90% (p < 0.001) relative to mock-transfected cells, there was a 20% downregulation (p < 0.05) of E2F1 TA (Figure 4C).

### Gene specific silencing of p73 associated with decreased p21 TA level

According to bivariate analysis shown in Figure 3, p73α and p21 were significantly correlated in cell lines that have inactivated p53. Therefore, we anticipated that gene specific silencing of p73 in one of these lines would directly reduce p21 TA. We used a pool of siRNAs to target all isoforms of p73 in the null-p53 small cell carcinoma N417. This cell line is an appropriate model because it expresses high p21 (34,000), E2F1 (19,000), and endogenous p73α (5,200) TA levels. p73α TA decreased by 80% relative to the non-specific siRNA control (p < 0.05), while p21 TA decreased by 70% (p < 0.01).

## Discussion

Relative to normal bronchial epithelial cells (BEC), p21 is upregulated in some bronchogenic carcinoma (BC) tissues and downregulated in others (Tables 1, 2, and 3). Elevation of p21 TA in malignant cells may seem counter-intuitive because it would be expected to slow cell proliferation. However, in some tumors pRb is dysfunctional and feedback signals such as p73 upregulation by E2F1 may increase p21 transcription in an ineffective attempt to prevent phosphorylation of pRb and release of activated E2F1. It is likely that such tumors would be poor candidates for therapy intended to control cell proliferation through specific upregulation of p21 transcription. However, in other tumors, such as those with a genetic profile similar to that of A549 or Calu-1 (Table 1), pRb is intact, and TA levels of E2F1, p73, and p21 are all low. In a tumor such as this, there is reason to believe that the upregulation of p21 transcription would be an effective treatment. However, in order to develop effective p21 gene-specific therapeutics, and biomarkers that predict which tumor will respond, it is necessary to better understand p21 transcriptional regulation.

In some cell types, E2F1 directly regulates p21 [28], however, the data presented here support the hypothesis that E2F1 does not upregulate p21 directly in human BC, but rather, elevated p21 TA results from E2F1 upregulation of p73. This hypothesis, generated initially from empirical observation, is supported by experimental data. There were four supportive empirical observations. First, there was lack of correlation between E2F1 and p21 TA among BC tissues. Second, there was positive correlation among BC tissues between E2F1 and p73 TA. Third, there was positive correlation between p73 and p21 TA among BC cell lines with inactivated p53. Fourth, in the H1155 BC cell line, in which p53 and p73 are inactivated, E2F1 and p73 TA levels were high, but p21 TA level was low. If E2F1 upregulated p21 directly, it would be reasonable to expect that p21 would be upregulated in this cell line, not downregulated.

In experiments designed to directly test this hypothesis, transient exogenous expression of p73α was associated with increased p21 and decreased E2F1 TA levels and siRNA mediated silencing of p73 was associated with decreased p21 TA levels. Although the siRNA experiments support our hypothesis, they are not as supportive as the transient transfection experiments because E2F1 TA was reduced along with p73 and p21. We speculate that this decrease was due to a non-specific effect of the siRNAs. A non-specific effect of the siRNA method has been previously reported for cell cycling genes, including p53 and p21 [36].

## Conclusion

In this study, we provide strong empirical and experimental evidence that in human bronchogenic carcinoma, p21 transcription is regulated by p73 but, in contrast to other cell types, not directly by E2F1. This knowledge will facilitate a) development of p21 gene-specific therapeutics necessary for individualized treatment strategies, and b) discovery of biomarkers that will predict which tumors will respond to p21 gene-specific therapeutics.

## Methods

### Normal cell populations

Normal BEC stock populations (lot numbers: 17378, 6F0333, 6F0395) were obtained from Clonetics (San Diego, CA) and incubated in BEGM medium.

### Carcinoma cell lines

Fourteen BC cell lines (Table 1) were obtained from American Type Culture Collection (Rockville, MD) and incubated in RPMI with 10% FBS.

### Culture conditions

Normal BEC and BC cells proliferate optimally under different conditions [37]. The medium that is optimal for BC cell lines, RPMI with 10% fetal bovine serum (FBS), induces terminal squamous differentiation in normal BEC [38]. In contrast, BC cell lines do not divide in serum-free media that are optimal for proliferation of normal BEC. BC cell lines were incubated in RPMI with 10% FBS and normal BEC from three individuals were incubated in bronchial epithelial growth medium (BEGM). In order to directly compare with carcinoma cell lines under the same optimal conditions, normal BEC cell populations (17378, 6F0333, 6F0395) were also incubated for 24 hours in RPMI with 10% FBS.

### Primary tissue samples

Primary normal BEC and primary BC samples (Table 2) were obtained under IRB approved protocols as previously described [29,39,40]. In each case informed consent was obtained from the patient.

### RNA extraction and reverse transcription

Total RNA was extracted by phenol/chloroform methods using TRI-Reagent (Molecular Research Center, Inc., Cincinnati, OH). Approximately 1 μg of total RNA was reverse transcribed using oligo dTs and MMLV-reverse transcriptase (Invitrogen, Inc., Carlsbad, CA).

### Transcript abundance measurement

Transcript abundance (TA) was measured by Standardized RT (StaRT)-PCR [30,41,42]. With this method there is an internal standard, within a standardized mixture of internal standards (SMIS) for each gene amplified in the PCR reaction. This enables regular assessment of performance characteristics as recommended by recent FDA guidelines [43]. Among these performance characteristics are reproducibility, lower detection threshold, linear dynamic range, signal to analyte response, false negatives, and false positives. For each gene measured in this study, the lower detection threshold was less than 10 molecules and the linear dynamic range was less than 10 to greater than 10^7 ^molecules. False negatives are eliminated due to the presence of an internal standard and false positives are eliminated by using a water control to ensure that there is no contamination within the PCR reaction.

The reagents for analysis of E2F1 and p21 were commercially prepared (Gene Express, Inc., Toledo, OH). To analyze p73, a SMIS containing internal standards for only p73 and β-actin were prepared in this laboratory, because p73 is not in a commercially available SMIS. p73 forward and reverse primers amplify at least four distinct isoforms including α-δ, but do not distinguish between the full-length and ΔN transcripts. A separate pair of primers published by Kartasheva, *et al*. [44] were used to determine the presence of ΔNp73. The internal standard for p73 was prepared using the forward primer and a competitive template (CT) primer. The CT primer hybridizes upstream of the reverse primer but retains its sequence at the 5' end [45]. This enables the simultaneous amplification of the internal standard and endogenous native template (NT) using only the forward and reverse primers. p73 forward and reverse primer sequences are as follows: p73 F, 5' ACT TTG AGA TCC TGA TGA AG 3' R, 5' CAG ATG GTC ATG CGG TAG TG 3'. Primer sequences for p21 and E2F1 were previously reported [30].

Six SMISs (A-F) were used for all TA measurements, with p73 CT at concentrations ranging from 10^-12 ^(SMIS A) to 10^-17 ^M (SMIS F) and β-actin CT constant at 10^-13 ^M in all six SMISs. The dilution of each cDNA sample that contained 60,000 molecules of β-actin cDNA was determined through calibration to 1 μL of SMIS F. Such calibrated samples were then used in all StaRT-PCR experiments. In some experiments, if the amount of cDNA sample available was low, the cDNA and the SMIS were both diluted 10-fold. Equal volumes of cDNA and SMIS were combined in a master mix along with 30 mM MgCl_2 _(Idaho Technology, Inc., Idaho Falls, ID), 2 mM dNTPs, Taq Polymerase (Promega, Madison, WI), and RNase-free water. For each TA measurement, a 10 μL reaction volume was PCR amplified in a Rapidcycler (Idaho Technology, Inc., Idaho Falls, ID) for 35 cycles. PCR reactions were denatured for 5 seconds at 94°C, annealed for 10 seconds at 58°C, and elongated for 15 seconds at 72°C.

### Plasmid DNA

A pcDNA 3.0 (Invitrogen Inc., Carlsbad, CA) expression vector was kindly provided from the laboratory of Vincenzo DeLaurenzi (University of Rome, Italy). The p73 gene contains a hemagglutinin tag sequence and is regulated by a CMV promoter sequence. An expression vector encoding a CMV regulated green fluorescent protein was obtained from (Gene Therapy Systems, San Diego, CA), and used as a negative control (mock) and determinant for transfection efficiency.

### Transient transfection assays

Calu-1 cells were incubated in RPMI supplemented with 10% FBS and grown to confluence. Twenty-four hours prior to transfection, cells were trypsinized and transferred to 60 mm dishes. For transfections, 5 μg of plasmid DNA was diluted in 0.5 mL of serum-free medium with the appropriate concentration of Lipofectamine 2000 transfection reagent (Invitrogen, Inc., Carlsbad, CA). Cells were incubated with DNA-lipid complexes in serum-containing medium for 4–8 hours and subsequently treated with fresh medium. RNA was isolated 24 hours post-transfection and analyzed by StaRT-PCR. 60 molecules of p73 internal standard were sufficient to quantify endogenous p73α TA and 6,000 molecules were required to quantify the combined endogenous and exogenous transcript. To exclude the possibility that the high level measured was partly due to amplification of contaminating plasmid DNA, RNA from p73α transfected Calu-1 cells was PCR-amplified with p73 specific primers. No PCR product was observed.

### Western blot analysis

Calu-1 cells were lysed 24 hours post-transfection by three consecutive freeze-thaws in a 0.25 M Tris lysis buffer (Invitrogen, Inc., Carlsbad, CA). Total protein concentration was determined colorimetrically using the bicinchoninic acid (BCA) assay (Pierce, Inc., Rockford, IL). 20 μg of total protein from Calu-1 cells were loaded on a 7% SDS Tris Acetate NuPage gel (Invitrogen, Inc., Carlsbad, CA). Proteins were transferred to a PVDF membrane and incubated with an anti-HA primary antibody conjugated to horseradish peroxidase (Santa Cruz Biotechnology, Santa Cruz, CA). The PVDF was then incubated with chemiluminescent substrates (Santa Cruz Biotechnology, Santa Cruz, CA) and visualized by autoradiography.

### siRNA (RNAi)

Approximately 1 million N417 cells were incubated in a six-well plate with RPMI supplemented with 10% FBS. Five siRNA oligonucleotides specific for the p73 gene or a non-specific pooled duplex control (Dharmacon, Inc., Lafayette, CO) were diluted in serum-free media and added to the appropriate concentration of TKO transfection reagent (Mirus Corp., Madison, WI). Untransfected cells were treated with transfection reagent but not siRNA. Transfected cells were incubated continuously with siRNA complexes at a final concentration of 100 nM. RNA was isolated 24 to 48 hours post-transfection and analyzed by StaRT-PCR.

### Bivariate and statistical analysis

Pearson correlation and paired-sample and independent T-tests were performed using SPSS 11.5.1 for Windows (SPSS, Chicago, IL) Due to inter-sample variation it was necessary to normalize the data by logarithmic transformation. For each test, a p-value of less than 0.05 was considered statistically significant. Bivariate graphs were created using Microsoft Excel 2000 (Microsoft Corp, Redmond, WA).

## Competing interests

ELC, KAW, and JCW each have significant equity interest in Gene Express, Inc., which produces and markets StaRT-PCR reagents used in these studies.

## Authors' contributions

MWH was responsible for TA measurement of p73 in all primary samples, TA measurement of E2F1 and p21 in primary samples, siRNA and transient transfection experiments, statistical analysis, and was the primary author of this manuscript. TGG was responsible for the preparation of the p73 SMIS, TA measurement of p73, E2F1, and p21 in all cultured cell lines, and contributed to the research design of this study. ELC, KAW, and CAMR were responsible for TA measurement of E2F1 and p21 and were involved in the acquisition and preparation of primary samples. JCW coordinated and obtained funding for this study and drafted and revised this manuscript. MWH, TGG, and JCW jointly conceived the experiments.
